# Living-donor transplantation leads to a major improvement in physical functioning: an observational study on the impact on potential donors and their recipients

**DOI:** 10.1186/s12882-019-1299-9

**Published:** 2019-03-29

**Authors:** Natascha J. H. Broers, Tsz Yeung Fung, Jeroen P. Kooman, Maarten H. L. Christiaans

**Affiliations:** 1 Department of Internal Medicine, Division of Nephrology, Maastricht University Medical Center+, PO Box 5800, 6202 AZ Maastricht, The Netherlands; 2 NUTRIM School of Nutrition and Translational Research in Metabolism, Maastricht University, Maastricht, The Netherlands

**Keywords:** Kidney transplantation, Living donor, Physical functioning, Physical activity, Body composition

## Abstract

**Background:**

Prospective studies combining physical functioning (PF), physical activity (PA), and body composition (BC) after living donor transplantation/donation are scarce. We aimed to study differences in these parameters between kidney transplant recipients and their living donors by examining changes in these parameters in the first post-operative year in both groups.

**Methods:**

Twenty-two kidney transplant recipients and 22 healthy kidney donors were included in this prospective longitudinal study with a follow-up until twelve months. PF was assessed by handgrip strength (HGS), and by the physical domains of health-related quality of life (HRQOL) using the Short Form-36 questionnaire [PF (SF-36 PF) and physical component summary (PCS) score]. BC was measured by the Body Composition Monitor©, and PA was measured by the SenseWear™ pro3.

**Results:**

At baseline, recipients had significantly lower HGS (after adjustment for sex and body weight), SF-36 PF, PCS, and PA, as compared with their donors. In recipients HGS significantly increased in the first year after transplantation, but PA did not change in the first six months after transplantation. Furthermore, no significant increase in lean tissue mass was observed. For healthy donors no significant changes in these parameters were observed, with exception of SF-36 PF, which declined in the first three months after donation, but equaled baseline values after twelve months.

**Conclusion:**

Recipients showed impressive improvements in PF and the physical domains of HRQOL in the first year after transplantation, reaching levels of healthy kidney donors already three to six months after transplantation. On the contrary, living kidney donation did not show any deterioration of the investigated parameters, supporting little impact for well-screened donors, while there is high benefit for transplant recipients.

## Background

Kidney transplantation (KTx) is the treatment of choice in end-stage renal disease (ESRD) patients, due to increased patient survival [1, 2], and better health-related quality of life (HRQOL) [3, 4]. Nevertheless, recurrent events such as graft failure and cardiovascular events affect long-term survival [2]. Decreased physical functioning (PF) and physical activity (PA), and concomitant weight gain in the first post-operative year after KTx are additional risk factors [5], and are associated with decreased patient survival [6]. Numerous studies showed that recipients gaining an average of almost 10 % body weight in the first post-operative year after KTx [7, 8], and that weight gain observed in the first months after KTx is predominantly due to an increase in fat mass [9]. Furthermore, weight gain is influenced by multiple factors such as age, food intake, basal metabolic rate, and PA [6, 10].

Studies combining different parameters related to the physical health domains are scarce. In addition, studies addressing PF, PA, body composition (BC) and HRQOL post-KTx [6, 11, 12] only focused on the early post-transplant outcomes (four months post-KTx) [13], used subjective measures for PA pre- and post-KTx [12], did not include healthy controls as comparators [14], or did not have parameters available before KTx [15].

Living donor transplantation provides the unique opportunity of studying the effects of a (near) reversal of the uremic state. Moreover, literature describing outcomes reflecting domains of PF and PA in living kidney donors are scarce as well [16], despite the fact that also in healthy donors these domains play an important role in the performance of activities of daily living and concomitant experienced HRQOL.

Therefore, we measured parameters reflecting PF, PA and BC in this study, and compared these parameters with their healthy donors. Furthermore, the parameters were assessed longitudinally during the first year after KTx and donation to consider the impact of the procedure in living donors and transplant recipients.

## Methods

**Fig. 1:**
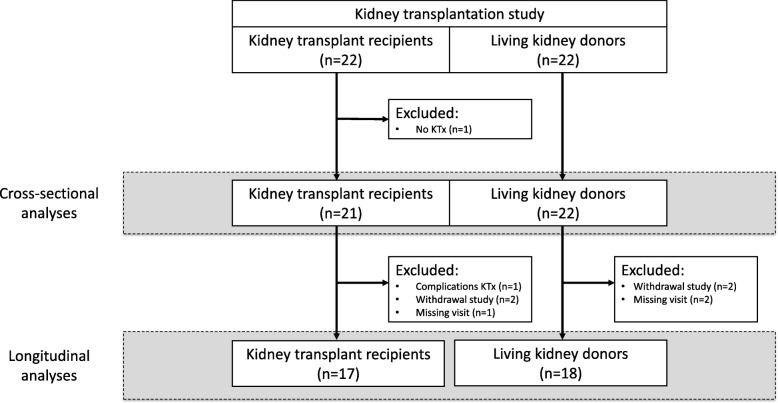
Recruitment and flow of participants; KTx = Kidney transplantation

This study was conducted between October 2013 and January 2018, and consisted of a cross-sectional part and a prospective longitudinal part. For this study 22 recipients and 22 donors were included, see Fig. 1 for the recruitment and flow of participants. Recipients and donors were recruited from the pre-transplantation clinic at the Maastricht University Medical Center+ in The Netherlands.

Inclusion criteria for recipients were: age ≥ 18 years, ability to provide informed consent, receiving a living donor kidney transplant. Not having an implantable cardioverter defibrillator (ICD) or pacemaker for bioimpedance measurements due to interference with the body composition monitor (BCM). Patients with an ICD or pacemaker had no restrictions for other measurements in the study.

Inclusion criteria for donors were: age ≥ 18 years, ability to provide informed consent, and suitable for living kidney donation, i.e. no uncontrolled or severe hypertension, and/or diabetes mellitus.

Recipients and donors were requested to be in a fasting state for the measurements, with exception of the PA measurements.

For each patient written informed consent was obtained prior to study participation. The Ethical Committee azM/UM (NL43381.068.13) and the Hospital Board of the Maastricht University Medical Center+ approved the study.

### Study design

**Fig. 2:**
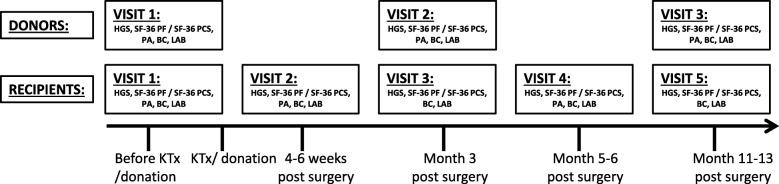
Overview of visits and measurements; HGS = handgrip strength, SF-36 PF = Short Form-36 physical functioning, SF-36 PCS = Short Form-36 physical component summary, PA = physical activity, BC = body composition, LAB = laboratory parameters, KTx = Kidney transplantation

This study included a cross-sectional and a longitudinal part. The methodology as described below has been described previously by our group [17, 18]. See Fig. 2 for an outline of the visits and measurements per visit. Visits were combined with regular pre- and post-operative visits at the out-patient transplant clinic or during period of hospitalization of the patient.

### Objectively measured physical functioning by muscle strength

Muscle strength, as an objective measure of PF, was determined with a hand held dynamometer (Jamar®, Sammons Preston Inc., Bolingbrook, IL). Mean handgrip strength (HGS) was determined in the dominant hand by measuring HGS in twofold. Measurements were taken in stand-up position with the arm flexed in a position of 90 degrees. For recipients who had already started hemodialysis (HD) therapy measurements were taken contralateral of the shunt arm.

### Self-reported physical functioning and physical activity related quality of life

Participants filled out Short Form-36 (SF-36) version 1 questionnaires to measure self-reported physical functioning (SF-36 PF) and the SF-36 physical component summary (PCS) score to evaluate the physical domains of HRQOL. Worldwide, the SF-36 is the most widely used questionnaire to assess HRQOL in the field of nephrology [19]. This multi-purpose short-form health survey, which includes 36 items, provides a measure of physical and mental health with a range from 0 (“worst possible health”) to 100 (“best possible health”). Questionnaires were scored using the algorithm of Ware et al. [20, 21]. T-score transformation was used to normalize scales (mean, 50 ± 10 [SD]) in order to make them comparable to the general population and other patients with specific diseases [21, 22].

### Physical activity measurements

To measure different parameters of PA [such as, total energy expenditure (TEE), activity related energy expenditure (AEE), number of steps] participants were asked to wear a SenseWear™ pro 3 armband (Bodymedia®, Pittsburg, PA) for two consecutive days. This is considered to be sufficient to measure daily physical activity [23, 24].The mean of the total on-body time was calculated for the analyses (which are showed as TEE, AEE and number of steps per 24 h). In addition, both TEE and AEE were expressed per kilogram body weight. Furthermore, we made no distinction between data collected on weekdays or weekends, both were included in the analyses for all participants.

### Body composition measurements

We determined BC by the use of bioimpedance spectroscopy with the Body Composition Monitor (BCM®, Fresenius Medical Care, Bad Homburg, Germany). The BCM uses a three-compartment model [adipose tissue mass (ATM), lean tissue mass (LTM), and a separate fluid overload (FO) compartment] as described by the model of Chamney et al. [25]. Measurements were performed as described by the manufacturer [26]. Patients were measured in supine position. For recipients who had already started dialysis therapy before KTx measurements were taken contralateral to the shunt arm in HD patients, or with a full abdomen in peritoneal dialysis (PD) patients for practical reasons. Moreover, it is shown that sequestered fluid in the trunk only has a little impact on whole-body bioimpedance measurements [27–29]. Furthermore, body weight was adjusted for PD fluid in the abdomen, as prescribed by the manufacturer [26]. Not all patients were in fasting state as requested for practical reasons e.g. diabetics. In addition, it has been shown that differences in pre- and postprandial BC estimates are small, and unlikely to be of clinical significance [30].

### Laboratory parameters

Laboratory parameters were determined during routine patient laboratory measurements. Estimated glomerular filtration rate (eGFR) was calculated by the CKD-EPI equation based on serum creatinine [31].

### Other clinical characteristics

Cause of renal disease, diabetes mellitus, and history of cardiovascular disease were based on the diagnosis as reported in the patient’s electronic health record. Office blood pressure was measured with an electronic sphygmomanometer (Omron M4-I, Omron, Japan).

### Statistical analysis

Data are expressed as mean ± SD or median [25th -75th percentile], unless indicated otherwise.

For the cross-sectional analyses, differences in the categorical variables were assessed by Chi-square tests. Differences in the continuous variables PCS scores, SF36-PF, HGS, PA, and BC between groups were examined with Mann-Whitney U tests as parameters were not normally distributed. Each cross-sectional analysis between recipients and healthy donors was based on all available data per parameter.

In an additional multivariable regression analysis, we adjusted the between-group differences in HGS for differences in the distribution of sex and body weight, since these parameters were unequally distributed between groups.

For the longitudinal analyses, comparison of changes over time within the recipient and the donor group were evaluated using Friedman tests, as most variables were not normally distributed. Each longitudinal analysis was based on complete cases per parameter (i.e. the recipient/donor was solely included into the analysis of a parameter if data was available for each time point).

All statistical analyses were performed with IBM SPSS Statistics for Windows, version 24 (IBM Corp. Armonk, NY, USA). *P*-values ≤0.05 were considered to be statistically significant.

## Results

### Patient characteristics

Baseline patient characteristics are summarized in Table 1. In addition, one patient was excluded from the study due to intercurrent disease before KTx (Fig. 1).

**Table 1:** Patient demographics

*Pre-surgery*	Recipients		Donors	
Number of Patients	21		22	
Male/Female (%)	47.6 / 52.4		36.4 / 63.6	
Age (years)	54.1 ± 12.5		55.5 ± 10.6	
Height (cm)	173.1 ± 10.4		171.6 ± 7.6	
Weight (kg)	73.8 ± 17.3		81.3 ± 15.7	
BMI (kg/m^2^)	24.5 ± 4.5		27.5 ± 4.6	
FO (L)	0.5 ± 1.8		−0.2 ± 1.3 (*n* = 21^c^)	
*Cause of end-stage renal disease*				
Diabetic nephropathy (%)	9.5 (*n* = 2)		-	
Polycystic kidney disease (%)	23.8 (*n* = 5)			
Nephrosclerosis (%)	4.8 (*n* = 1)			
Hypertensive nephropathy (%)	19.0 (*n* = 4)			
IgA nephropathy (%)	14.3 (*n* = 3)			
Unknown cause (%)	4.8 (*n* = 1)			
Other (%)	23.8 (*n* = 5)			
Diabetes Mellitus (%)	14.3 (*n* = 3)		0	
Cardiovascular Disease (%)^a^	14.3 (*n* = 3)		13.6 (*n* = 3)^b^	
eGFR (ml/min/1.73m^2^)	8.7 ± 3.6		85.7 ± 11.8	
Albumin (g/L)	36.7 ± 4.7		40.4 ± 4.0 (*n* = 20^c^)	
C-reactive protein (mg/L)	5.0 ± 5.7		2.7 ± 3.2 (*n* = 20^c^)	
Hemoglobin (mmol/L)	7.5 ± 0.9		8.9 ± 0.9 (*n* = 16^c^)	
Total CO2 (mmol/L)	27.1 ± 2.9		–	
Previous transplant (%)	14.3 (*n* = 3)		–	
Pre-emptive transplant (%)	42.9 (*n* = 9)		–	
Pre-KTx HD/PD	6/6		–	
SBP (mmHg)	149.6 ± 24.4		140.3 ± 19.9	
DBP (mmHg)	86.3 ± 12.4		86.8 ± 12.3	
*Post-surgery*				
eGFR (ml/min/1.73m^2^)	(*n* = 17^c^)			
1 month post-surgery	45.1 ± 11.5		–	
3 months post-surgery	48.1 ± 10.4		58.8 ± 11.1 (*n* = 18^c^)	
6 months post-surgery	52.2 ± 9.6		–	
12 months post-surgery	53.9 ± 8.7		54.5 ± 8.8 (*n* = 15^c^)	
Hemoglobin (mmol/L)	(*n* = 16^c^)		n/a	
1 month post-surgery	6.8 ± 1.3			
3 months post-surgery	7.5 ± 1.2			
6 months post-surgery	8.2 ± 1.1			
12 months post-surgery	7.9 ± 2.2			
Blood pressure (mmHg)	SBP	DBP	SBP	DBP
	(*n* = 17^c^)	(*n* = 17^c^)	(*n* = 18^c^)	(*n* = 18^c^)
1 month post-surgery	148.6 ± 25.9	86.9 ± 11.8	–	–
3 months post-surgery	134.2 ± 21.1	84.5 ± 15.4	132.7 ± 21.5	87.1 ± 11.1
6 months post-surgery	139.8 ± 16.7	86.2 ± 14.0	–	–
12 months post-surgery	138.5 ± 16.4	82.6 ± 11.2	135.6 ± 27.1	86.7 ± 12.8

Data are presented as mean ± SD

BMI body mass index, *FO* fluid overload, *SBP* systolic blood pressure, *DBP* diastolic blood pressure, *eGFR* estimated glomerular filtration rate

^a^Hypertension is excluded, ^b^CVD diagnoses in kidney transplant donors included infrarenal aortic abdominal aneurysm (*n* = 1), and transient ischemic attack (*n* = 2), ^c^ Data available in number of patients (*n*=)

### Immunosuppressive protocol

The main features of our center immunosuppressive protocol were: all patients received from time of transplant tacrolimus (TAC) and mycophenolate mofetil (MMF). In addition, all recipients received corticosteroids for ten days. Prednisolone was only continued or re-introduced in recipients with a high risk for rejection or in patients with an IgA Nephropathy as primary disease.

After three months MMF was stopped in patients with low or intermediate immunological risk and without rejection in the first three months, and a normal protocol biopsy at month three. In addition, three patients included in this study were simultaneously enrolled in a different study (TRANSFORM) (Identifier Clinicaltrials.gov: NCT01950819). Two of these three patients were randomized to receive TAC, everolimus and prednisolone as triple therapy from time of transplant. One of these two patients withdrew consent for our study in the first week after KTx due to personal reasons. The other patient withdrew consent for the TRANSFORM study due to personal reasons; this patient was converted to TAC based dual-therapy after the six month follow up visit of our study. The third patient was randomized to receive TAC, MMF and prednisolone as triple therapy from time of transplant.

Twelve months post-KTx seven patients were on TAC-based monotherapy, six patients on TAC based dual-therapy and five patients on TAC-based triple therapy.

### Rejections

During the twelve month follow-up period of this study four patients were treated for rejection. All received methylprednisolone. In addition, two patients received intravenous immunoglobulin because of a humoral rejection component.

### Kidney function

At baseline eGFR was 8.7 ± 3.6 ml/min/1.73m^2^ in the recipient group, and 85.7 ± 11.8 ml/min/1.73m^2^ in the donor group. At the twelve month follow-up visit eGFR increased to 53.9 ± 8.7 ml/min/1.73m^2^ in the recipient group. In the donor group eGFR declined to 54.5 ± 8.8 ml/min/1.73m^2^ twelve months post-donation, comparable to eGFR values of the recipient group at twelve months post-KTx (Table 1).

### Muscle strength

In the cross-sectional analysis no statistically significant difference was found for HGS (Table 2), whereas after adjustment for differences in sex and body weight HGS was significantly lower in the recipient group before KTx, as compared with that of the healthy donor group (− 4.7 kg, 95% CI -9.3 to − 0.1; *p* = 0.047).

**Table 2:** Cross-sectional analyses of parameters of physical functioning and physical activity

Cross-sectional analyses Recipients vs. Donors (baseline)	
*Handgrip strength (kg)*	
Recipient (n = 20)	24.3 [20.0–39.0]
Donor (*n* = 22)	29.5 [23.0–37.8]
p-value	0.378^a^
*SF-36 PCS score (%)*	
Recipient (n = 21)	46.3 [38.1–51.2]
Donor (*n* = 22)	56.3 [54.3–57.8]
p-value	< 0.001
*SF-36 Physical functioning (%)*	
Recipient (n = 21)	46.1 [41.8–52.5]
Donor (n = 22)	56.8 [52.0–56.8]
p-value	< 0.001
*Number of Steps/day* ^*b*^	
Recipient (n = 15)	6003.0 [3608.0–10,429.0]
Donor (*n* = 14)	12,711.0 [9460.5–15,194.0]
p-value	0.004
*TEE/kg/day* ^*−1 b*^	
Recipient (n = 15)	29.4 [26.5–34.2]
Donor (n = 14)	32.7 [31.0–37.7]
p-value	0.063
*AEE/kg/day* ^*−1b*^	
Recipient (n = 15)	2.3 [1.0–8.7]
Donor (n = 14)	7.6 [5.7–9.9]
p-value	0.016

Data are presented as median [25th and 75th percentile]

*SF* short form, *TEE* total energy expenditure, *AEE* activity related energy expenditure

^a^After adjustment for differences in sex and bodyweight *p* = 0.047, ^c^ Data available in 15 recipients/14 donors

**Fig. 3:**
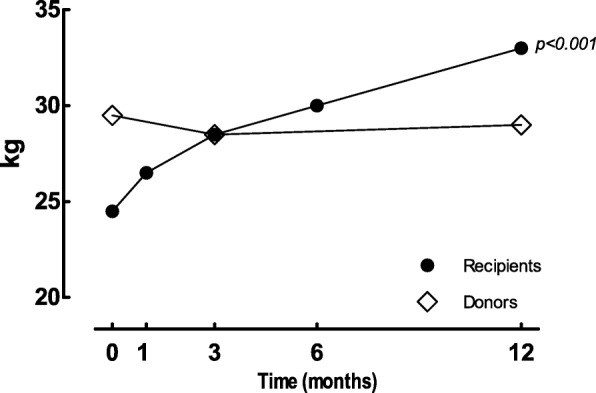
Handgrip strength. Data are presented as median

In the longitudinal analysis HGS significantly changed in the first year after KTx (*p* < 0.001), with values comparable to their donors by three months post-KTx. (Table 3, Fig. 3). No significant changes in HGS were found over time in the donor group, where twelve month HGS values were equal to pre-donation values.

**Table 3:** Longitudinal analyses of parameters of physical functioning and physical activity

Parameter	Recipients	Donors
*Handgrip strength (kg)*		
Baseline	24.5 [20.5–40.0]	29.5 [23.0–41.8]
1 month post-surgery	26.5 [19.0–41.3]	–
3 months post-surgery	28.5 [22.8–41.5]	28.5 [22.4–46.9]
6 months post-surgery	30.0 [24.3–41.0]	–
12 months post-surgery	33.0 [25.0–41.0]	29.0 [25.0–39.4]
p-value	< 0.001 (n = 17)	0.986 (n = 18)
*SF-36 PCS score (%)*		
Baseline	45.1 [37.7–51.2]	56.7 [54.9–57.8]
1 month post-surgery	33.7 [30.5–43.1]	–
3 months post-surgery	44.8 [34.3–52.1]	55.3 [48.8–57.9]
6 months post-surgery	52.2 [47.9–54.6]	–
12 months post-surgery	52.1 [48.5–56.3]	55.2 [50.4–59.0]
p-value	< 0.001 (n = 17)	0.234 (n = 18)
*SF-36 Physical Functioning (%)*		
Baseline	46.1 [41.8–52.5]	56.8 [54.1–56.8]
1 month post-surgery	41.8 [35.3–50.3]	-
3 months post-surgery	50.3 [41.8–54.6]	54.6 [50.9–56.8]
6 months post-surgery	54.6 [50.3–54.6]	–
12 months post-surgery	54.6 [52.5–54.6]	54.6 [52.5–56.8]
p-value	< 0.001 (n = 17)	0.049 (n = 18)
*Number of Steps/day* ^*a*^		
Baseline	6599.0 [4459.5–11,320.0]	13,287.0 [9917.0–14,731.0]
3 months post-surgery	–	11,513.0 [9839.0–14,429.0]
6 months post-surgery	7042.0 [5198.5–10,078.5]	–
12 months post-surgery	–	12,773.0 [9243.0–18,508.0]
p-value	0.917 (*n* = 13)	0.529 (*n* = 11)
*TEE/kg/day* ^*−1 a*^		
Baseline	31.2 [26.8–35.3]	32.8 [31.1–37.7]
3 months post-surgery	–	32.8 [28.9–39.9]
6 months post-surgery	31.6 [29.5–36.1]	–
12 months post-surgery	–	33.2 [29.1–38.8]
p-value	0.552 (n = 13)	0.850 (n = 11)
*AEE/kg/day* ^*−1a*^		
Baseline	4.7 [0.9–9.3]	9.0 [5.9–10.6]
3 months post-surgery	–	5.7 [4.8–15.0]
6 months post-surgery	3.4 [2.6–6.9]	–
12 months post-surgery	–	5.4 [4.8–14.5]
p-value	0.972 (n = 13)	0.486 (n = 11)

Data are presented as median [25th and 75th percentile]

*TEE* total energy expenditure, *AEE* activity related energy expenditure, *SF* short form

^a^Data available in 13 recipients/11 donors

### Self-reported physical functioning and physical activity related quality of life

In the cross-sectional analyses SF-36 PF and the PCS score, both measured by the SF-36, were significantly lower in the recipient group as compared with the healthy donor group (*p* < 0.001) (Table 2).

**Fig. 4:**
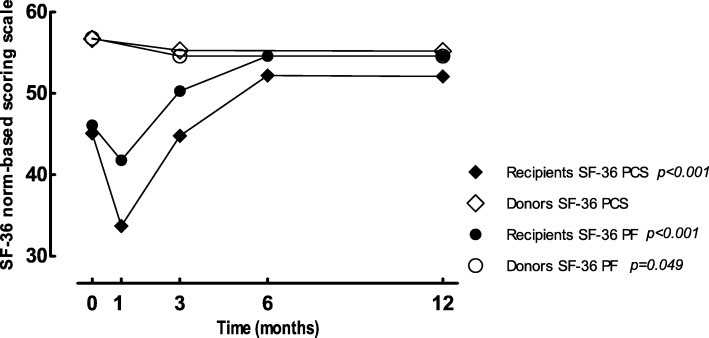
Physical domains of the Short Form-36 scores; SF-36 PCS = Short Form-36 physical component summary, SF-36 PF = Short Form-36 physical functioning. Data are presented as median

In the recipient group SF-36 PF and the PCS score did significantly increase in the first year after KTx (*p* < 0.001), and reached values approaching those of healthy donors six months after KTx (Table 3, Fig. 4). No significant changes over time were observed in the donor group for PCS scores (Table 3, Fig. 4). SF-36 PF statistically significantly declined in the first year after donation (median ∆SF-36 PF: 0.0 [− 2.1–0.0], *p* = 0.049). Although values were borderline significant, no clinically significant decline was observed in SF-36 PF (Table 3, Fig. 4).

### Physical activity

In the cross-sectional analysis, the average monitoring period with the sensewear armband was 1.92 ± 0.43 days with a mean on-body time of 97.7% in the recipient group, and 1.86 ± 0.92 days with a mean on-body time of 97.7% in the donor group.

In the longitudinal analysis of the recipient group the average monitoring period with the sensewear armband was 1.92 ± 0.25 days with a mean on-body time of 97.3% prior to KTx, and 1.78 ± 0.51 days with a mean on-body time of 98.7% six months after KTx. In the donor group the average monitoring period with the sensewear armband was 1.91 ± 1.02 days with a mean on-body time of 98.1% prior to donation, 1.84 ± 0.54 days with a mean on-body time of 94.2% three months after donation, and 2.07 ± 0.46 days with a mean on-body time of 92.8% twelve months after donation.

Number of steps were significantly lower in ktx recipients as compared with those in healthy donors (*p* = 0.003) (Table 2). AEE was also significantly lower as compared with healthy donors (*p* = 0.016), and TEE showed a clear tendency to significance (*p* = 0.063) (Table 2).

Furthermore, the longitudinal analyses showed that in the first six months after KTx number of steps did not significantly increase over time in the recipient group (Table 3). Likewise, in the donor group no significant changes in PA parameters were found in the first year after donation (Table 3).

### Body composition

At baseline, statistically significant differences in BC were found between recipients and healthy donors in ATM, which was significantly lower in the recipient group; (median 31.8 kg [21.0;39.0]) vs. (35.0 kg [26.4;50.6]) (*p* = 0.044), and FO which was significantly higher in the recipient group; (median 0.9 L [− 0.1;1.5]) vs. (− 0.3 L [− 0.9;0.4]) (*p* = 0.005), respectively.

**Fig. 5:**
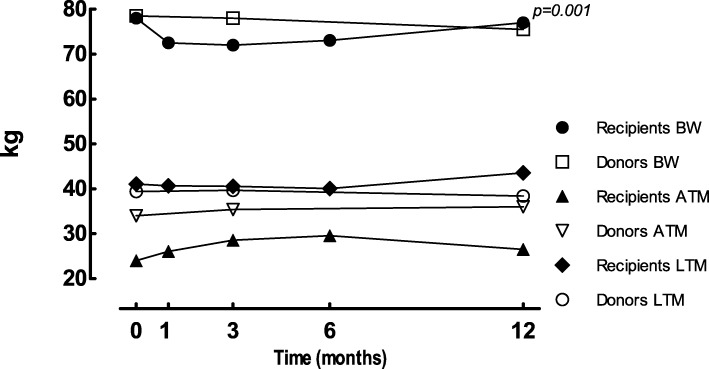
Body composition; BW = body weight, ATM = adipose tissue mass, LTM = lean tissue mass. Data are presented as median

Significant changes over time were found in the recipient group for BC parameters body weight (*p* = 0.001) (Table 4, Fig. 5), BMI (p = 0.001), and FO (*p* = 0.032) (Table 4). No changes in BC were found in the healthy donor group in the first year after donation (Table 4, Fig. 5).

**Table 4:** Longitudinal analyses of body composition parameters

Parameter	Recipients	Donors
*Body weight (kg)*		
Baseline	78.0 [59.4–89.0]	78.5 [71.4–92.4]
1 month post-surgery	72.5 [58.6–85.8]	–
3 months post-surgery	72.0 [59.6–87.0]	78.0 [70.3–92.8]
6 months post-surgery	73.1 [60.0–89.0]	–
12 months post-surgery	77.0 [61.0–90.5]	75.5 [71.5–95.3]
p-value	0.001 (n = 17)	0.554 (n = 18)
*BMI (kg/m* ^*2*^ *)*		
Baseline	24.4 [21.5–28.7]	26.4 [23.0–29.6]
1 month post-surgery	24.2 [20.5–26.6]	–
3 months post-surgery	24.5 [20.6–26.9]	26.1 [22.9–28.5]
6 months post-surgery	23.8 [21.5–27.3]	–
12 months post-surgery	23.8 [21.3–28.6]	26.3 [22.6–28.7]
p-value	0.001 (*n* = 17)	0.554 (*n* = 18)
*LTM (kg)*		
Baseline	41.1 [36.4–59.7]	39.4 [35.0–50.9]
1 month post-surgery	40.7 [35.8–47.2]	–
3 months post-surgery	40.6 [33.8–58.9]	39.7 [34.2–47.7]
6 months post-surgery	40.1 [36.0–53.2]	–
12 months post-surgery	43.6 [34.8–53.5]	38.4 [34.3–52.1]
p-value	0.173 (n = 15^a^)	0.200 (n = 18)
*ATM (kg)*		
Baseline	24.0 [20.6–39.5]	34.0 [26.6–50.3]
1 month post-surgery	26.1 [17.1–40.8]	–
3 months post-surgery	28.6 [19.6–36.4]	35.4 [27.2–48.0]
6 months post-surgery	29.6 [21.5–38.1]	–
12 months post-surgery	26.5 [19.6–39.7]	36.0 [26.4–44.3]
p-value	0.283 (*n* = 15^a^)	0.504 (n = 18)
*FO (L)*		
Baseline	0.9 [−0.1–1.2]	−0.3 [−1.0–0.5]
1 month post-surgery	1.7 [0.4–2.5]	–
3 months post-surgery	1.2 [0.2–1.7]	−0.3 [− 0.7–0.3]
6 months post-surgery	0.7 [− 0.2–1.7]	–
12 months post-surgery	0.5 [− 0.1–1.0]	−0.6 [− 1.1–0.2]
p-value	0.032 (n = 16^a^)	0.689 (n = 18)

Data are given as median [25th and 75th percentile]

*BMI* body mass index, *LTM* lean tissue mass, *ATM* adipose tissue mass, *FO* fluid overload

^a^Data available in number of patients (n=)

## Discussion

This study showed impressive improvements in PF, assessed by HGS and the SF-36, during the first year after KTx with a living donor. Values of these parameters already approached those of their healthy controls three to six months after KTx. These improvements were not paralleled by comparable changes in BC and PA. Furthermore, this study showed no significant deterioration of any of the parameters investigated in healthy donors in the first year after living kidney donation.

To the best of our knowledge this is the first study that included a cohort of healthy donors as a control group to study differences between kidney transplant patients and their living donors. In addition, this is the first study which examined changes in parameters of PF and PA both objectively (by HGS and accelerometry), and subjectively (by patient reported outcomes: SF-36 questionnaires) in both patient groups in the first twelve months after living donor transplantation. These outcomes give a unique insight into the impact of the procedure in both the transplant recipient, and the living kidney donor.

Results at baseline showed that recipients had lower levels of PF (as subjectively assessed by SF-36 questionnaire, and objectively assessed by HGS measurements), PCS scores, and PA as compared with the healthy donor group prior to the KTx/donation. Regarding BC; body weight and LTM were not significantly different at baseline in the recipient group as compared with the healthy donors. ATM was significantly higher in the healthy donor group, and FO was significantly higher in the recipient group prior to the KTx/donation.

Longitudinal analyses in the healthy donor group showed a significant reduction of residual renal function twelve months after donation to an eGFR of 54.5 ± 8.8 ml/min/1.73m^2^. However, the decrease in eGFR was not paralleled by a significant decrease in parameters of PF, PA and BC in the first year after donation, with exception for SF-36 PF, which statistically significantly declined in the first three months. Nevertheless, twelve months post-donation it approached baseline values again. Interestingly, in transplant recipients, an increase in eGFR to 53.9 ± 8.7 ml/min/1.73m^2^ in the first twelve months after KTx, led to significant improvements in parameters of PF and the physical domains of HRQOL. With values comparable to baseline values of their healthy donors, despite the fact that these improvements were not paralleled by an increase in PA or LTM.

The significant improvement in HGS in the recipient group after KTx is in line with a study of Lorenz et al. that showed increased HGS four months after KTx as well [13]. Although LTM decreased after KTx, and did not reach baseline values until twelve months post-KTx, possibly due to catabolic effects of surgery itself or reduced dietary intake in the early post-transplant period, an increase in muscle strength in the early post-KTx period was observed. This suggests that other factors might be responsible for the increase in muscle strength such as reversal of uremic myopathy after KTx due to the reversal of the uremic state. This is supported by findings of Painter et al. who showed an increase in muscle strength in the first year after KTx irrespective of receiving exercise interventions or usual patient care [32]. Furthermore, the steroid-sparing immunosuppressive protocol in our center might contribute to positive effects on PF as well. Earlier studies have already described significant improvements in muscle structure, which could contribute to better muscle function and exercise capacity, in KTx patients with a steroid-free maintenance immunosuppression regimen [33].

As a subjective marker of PF, SF-36 PF and PCS scores were measured. Despite the fact that both scores were significantly lower before KTx as compared with scores of their healthy donors, scores were approaching reference values within six months after KTx [34]. In addition, this increase in PCS and SF-36 PF scores after KTx is in line with other studies [35].

The near normalization of PF after KTx, as assessed by the parameters described earlier, is in contrast to earlier findings including those of our own group, which showed lower levels of PF and performance in KTx patients as compared with healthy controls [36]. A possible explanation could be that we used only relatively simplified measures in the present study such as dynamometrically measured HGS and patient-reported outcomes of PF, as compared to those of others who used peak oxygen uptake (VO_2_ peak) and dynamometric measures of lower extremity function [36, 37]. However, it could be also due to the fact that in our recipient group a near full normalization of renal function was achieved. Interestingly, although the relation between PA and physical performance scores in uremic patients is well established [11], we did not observe changes in PA that paralleled changes in PF. Admittedly, in our study protocol PA was only assessed at baseline and six months after KTx, albeit at that time point impressive improvements in PF were already observed. Although the literature is still contradictory concerning the improvement of PA after KTx [15, 38], it is well described that daily PA recommendations are not reached by the majority of KTx recipients [12, 39, 40].

In the healthy donor group the PCS score did not change in the first year after donation. For SF-36 PF the minor decline in the first three months after donation is most likely due to post-operative outcomes. Nevertheless, scores still remained higher than scores of the general population [34], and equaled baseline values after twelve months. These findings are in line with recent studies of Janki et al. [41, 42], which support our findings that there are no negative short-term consequences for donors with regard to the physical domains of HRQOL.

Furthermore, short-term results showed that HGS, parameters of PA, and BC did not change in the first year after donation, despite an approximately 35% decrease in eGFR. These findings support the safety of living kidney donation as well. Although long-term effects still have to be investigated on these domains, short-term changes in terms of PF and PA support a relatively small impact for the healthy donor in contrast to the major benefits for the recipient. Given the fact that a majority of the living kidney donors are related to the recipient, the psychosocial feeling with regard to the donors’ self-esteem and satisfaction after donation are often very positive showing no evidence of harm as well [43].

Some drawbacks deserve consideration: first, the relatively small study population. Nevertheless, comparable studies did not include the pre-transplant period as baseline measures or did not use objective measures as outcome, as discussed previously. Second, as mentioned previously, the design of our protocol included only the assessment of PA at baseline and six months thereafter. Third, we aimed to provide a multidimensional spectrum, though we were only able to include a selected number of parameters for each domain. Admittedly, some measurements, such as MRI, are far more sensitive to assess changes in LTM as compared to bioimpedance spectroscopy. However, we believe that the broad spectrum of measurements balanced out the increased precision of a single more specific measurement.

## Conclusions

In conclusion, PF and the physical domains of HRQOL showed impressive improvements in the first year after KTx, reaching levels comparable with healthy kidney donors already three to six months after living KTx, while living kidney donation did not result in any deterioration of the investigated parameters. The outcomes showed relatively minor impact for well-screened donors. With on the opposite high benefits in a relative short period of time for the transplant recipient in terms of regaining levels of PF and HRQOL, as compared with reference values of their healthy donors and the general population, supporting the possibility of living kidney transplantation.

## Abbreviations

∆: delta
AEE: Activity related energy expenditure
ATM: Adipose tissue mass
BC: Body composition
BCM: Body composition monitor
ESRD: End-stage renal disease
FO: Fluid overload
GFR: Glomerular filtration rate
HD: Hemodialysis
HGS: Handgrip strength
HRQOL: Health-related quality of life
ICD: Implantable cardioverter defibrillator
KTx: Kidney transplantation
LTM: Lean tissue mass
MMF: Mycophenolate mofetil
PA: Physical activity
PCS: Physical component summary
PD: Peritoneal dialysis
PF: Physical functioning
SD: Standard deviation
SF-36 PF: Short Form-36 physical functioning
SF-36: Short Form-36
TAC: Tacrolimus
TEE: Total energy expenditure

## References

[1] Wolfe RA, Ashby VB, Milford EL, Ojo AO, Ettenger RE, Agodoa LY, Held PJ, Port FK (1999). Comparison of mortality in all patients on dialysis, patients on dialysis awaiting transplantation, and recipients of a first cadaveric transplant. N Engl J Med.

[2] Kasiske BL (2001). Epidemiology of cardiovascular disease after renal transplantation. Transplantation.

[3] Maglakelidze N, Pantsulaia T, Tchokhonelidze I, Managadze L, Chkhotua A (2011). Assessment of health-related quality of life in renal transplant recipients and dialysis patients. Transplant Proc.

[4] Overbeck I, Bartels M, Decker O, Harms J, Hauss J, Fangmann J (2005). Changes in quality of life after renal transplantation. Transplant Proc.

[5] Abecassis M, Bridges ND, Clancy CJ, Dew MA, Eldadah B, Englesbe MJ, Flessner MF, Frank JC, Friedewald J, Gill J (2012). Solid-organ transplantation in older adults: current status and future research. Am J Transplant.

[6] Heng AE, Montaurier C, Cano N, Caillot N, Blot A, Meunier N, Pereira B, Marceau G, Sapin V, Jouve C (2015). Energy expenditure, spontaneous physical activity and with weight gain in kidney transplant recipients. Clin Nutr.

[7] Orazio L, Chapman J, Isbel NM, Campbell KL (2014). Nutrition care for renal transplant recipients: an evaluation of service delivery and outcomes. J Ren Care.

[8] Armstrong KA, Campbell SB, Hawley CM, Johnson DW, Isbel NM (2005). Impact of obesity on renal transplant outcomes. Nephrology (Carlton).

[9] van den Ham EC, Kooman JP, Christiaans MH, Leunissen KM, van Hooff JP (2000). Posttransplantation weight gain is predominantly due to an increase in body fat mass. Transplantation.

[10] Rimbert V, Montaurier C, Bedu M, Boirie Y, Morio B (2006). Behavioral and physiological regulation of body fatness: a cross-sectional study in elderly men. Int J Obes.

[11] Raymond J, Johnson ST, Diehl-Jones W, Vallance JK (2016). Walking, sedentary time and health-related quality life among kidney transplant recipients: an exploratory study. Transplant Proc.

[12] Nielens H, Lejeune TM, Lalaoui A, Squifflet JP, Pirson Y, Goffin E (2001). Increase of physical activity level after successful renal transplantation: a 5 year follow-up study. Nephrol Dial Transplant.

[13] Lorenz EC, Cheville AL, Amer H, Kotajarvi BR, Stegall MD, Petterson TM, Kremers WK, Cosio FG, LeBrasseur NK. Relationship between pre-transplant physical function and outcomes after kidney transplant. Clin Transpl. 2017;31(5). 10.1111/ctr.12952. 10.1111/ctr.12952 PMC5416778 28295612

[14] Pantik C, Cho YE, Hathaway D, Tolley E, Cashion A. Characterization of body composition and fat mass distribution 1 year after kidney transplantation. Prog Transplant. 2017;27(1):10–15. 10.1177/1526924816681007 PMC6086117 27903767

[15] Dontje ML, de Greef MH, Krijnen WP, Corpeleijn E, Kok T, Bakker SJ, Stolk RP, van der Schans CP (2014). Longitudinal measurement of physical activity following kidney transplantation. Clin Transpl.

[16] Suwelack B, Wormann V, Berger K, Gerss J, Wolters H, Vitinius F, Burgmer M, Lc GS (2018). Investigation of the physical and psychosocial outcomes after living kidney donation - a multicenter cohort study (SoLKiD - safety of living kidney donors). BMC Nephrol.

[17] Broers NJH, Martens RJH, Cornelis T, van der Sande FM, Diederen NMP, Hermans MMH, Wirtz J, Stifft F, Konings C, Dejagere T (2017). Physical activity in end-stage renal disease patients: the effects of starting Dialysis in the first 6 months after the transition period. Nephron.

[18] Broers NJH, Martens RJH, Canaud B, Cornelis T, Dejagere T, Diederen NMP, Hermans MMH, Konings C, Stifft F, Wirtz J (2018). Health-related quality of life in end-stage renal disease patients: the effects of starting dialysis in the first year after the transition period. Int Urol Nephrol.

[19] Wight JP, Edwards L, Brazier J, Walters S, Payne JN, Brown CB (1998). The SF36 as an outcome measure of services for end stage renal failure. Qual Health Care.

[20] Ware JE, Snow KK, Kosinski M, Gandek B (1993). Health survey manual and interpretation guide.

[21] Ware JE, Kosinski M, Keller SD (1994). SF-36 Physical and Mental Health Summary Scales: A User's Manual, ed 2.

[22] Ware JE (2001). SF-36 health survey update. SPINE.

[23] Dontje ML, van der Wal MH, Stolk RP, Brugemann J, Jaarsma T, Wijtvliet PE, van der Schans CP, de Greef MH (2014). Daily physical activity in stable heart failure patients. J Cardiovasc Nurs.

[24] Almeida GJ, Wasko MC, Jeong K, Moore CG, Piva SR (2011). Physical activity measured by the SenseWear armband in women with rheumatoid arthritis. Phys Ther.

[25] Chamney PW, Wabel P, Moissl UM, Muller MJ, Bosy-Westphal A, Korth O, Fuller NJ (2007). A whole-body model to distinguish excess fluid from the hydration of major body tissues. Am J Clin Nutr.

[26] Fresenius Medical Care (2007). Body Composition Monitor Manual Operation Procedure.

[27] Van Biesen W, Williams JD, Covic AC, Fan S, Claes K, Lichodziejewska-Niemierko M, Verger C, Steiger J, Schoder V, Wabel P (2011). Fluid status in peritoneal dialysis patients: the European body composition monitoring (EuroBCM) study cohort. PLoS One.

[28] Davison SN, Jhangri GS, Jindal K, Pannu N (2009). Comparison of volume overload with cycler-assisted versus continuous ambulatory peritoneal dialysis. Clin J Am Soc Nephrol.

[29] Cooper BA, Aslani A, Ryan M, Zhu FY, Ibels LS, Allen BJ, Pollock CA (2000). Comparing different methods of assessing body composition in end-stage renal failure. Kidney Int.

[30] Androutsos O, Gerasimidis K, Karanikolou A, Reilly JJ, Edwards CA (2015). Impact of eating and drinking on body composition measurements by bioelectrical impedance. J Hum Nutr Diet.

[31] Levey AS, Stevens LA, Schmid CH, Zhang YL, Castro AF, Feldman HI, Kusek JW, Eggers P, Van Lente F, Greene T (2009). A new equation to estimate glomerular filtration rate. Ann Intern Med.

[32] Painter PL, Hector L, Ray K, Lynes L, Dibble S, Paul SM, Tomlanovich SL, Ascher NL (2002). A randomized trial of exercise training after renal transplantation. Transplantation.

[33] Topp KS, Painter PL, Walcott S, Krasnoff JB, Adey D, Sakkas GK, Taylor J, McCormick K, TeNyenhuis M, Iofina M (2003). Alterations in skeletal muscle structure are minimized with steroid withdrawal after renal transplantation. Transplantation.

[34] Mols F, Pelle AJ, Kupper N (2009). Normative data of the SF-12 health survey with validation using postmyocardial infarction patients in the Dutch population. Qual Life Res.

[35] Villeneuve PM, Clark EG, Sikora L, Sood MM, Bagshaw SM (2016). Health-related quality-of-life among survivors of acute kidney injury in the intensive care unit: a systematic review. Intensive Care Med.

[36] van den Ham EC, Kooman JP, Schols AM, Nieman FH, Does JD, Franssen FM, Akkermans MA, Janssen PP, van Hooff JP (2005). Similarities in skeletal muscle strength and exercise capacity between renal transplant and hemodialysis patients. Am J Transplant.

[37] Painter P, Hanson P, Messer-Rehak D, Zimmerman SW, Glass NR (1987). Exercise tolerance changes following renal transplantation. Am J Kidney Dis.

[38] Cashion AK, Hathaway DK, Stanfill A, Thomas F, Ziebarth JD, Cui Y, Cowan PA, Eason J (2014). Pre-transplant predictors of one yr weight gain after kidney transplantation. Clin Transpl.

[39] Lin SY, Fetzer SJ, Lee PC, Chen CH (2011). Predicting adherence to health care recommendations using health promotion behaviours in kidney transplant recipients within 1-5 years post-transplant. J Clin Nurs.

[40] Rosas SE, Reese PP, Huan Y, Doria C, Cochetti PT, Doyle A (2012). Pretransplant physical activity predicts all-cause mortality in kidney transplant recipients. Am J Nephrol.

[41] Janki S, Dols LF, Timman R, Mulder EE, Dooper IM, van de Wetering J, IJzermans JN (2017). Five-year follow-up after live donor nephrectomy - cross-sectional and longitudinal analysis of a prospective cohort within the era of extended donor eligibility criteria. Transpl Int.

[42] Janki S, Klop KW, Dooper IM, Weimar W, Ijzermans JN, Kok NF (2015). More than a decade after live donor nephrectomy: a prospective cohort study. Transpl Int.

[43] Maple H, Chilcot J, Weinman J, Mamode N (2017). Psychosocial wellbeing after living kidney donation - a longitudinal, prospective study. Transpl Int.

